# Supplementary material for: Identification of photoperiod-induced specific miRNAs in the adrenal glands of Sunite sheep (Ovis aries)
Source: Front Vet Sci. 2022 Jul 22;9:888207. doi: 10.3389/fvets.2022.888207 (PMC9354845; doi:10.3389/fvets.2022.888207)
Supplement: Supplementary file 1 [file Data_Sheet_1.ZIP › Supplementary file/miRDeep2 file.zip!/miRDeep2 file/Know_Structure/SP42A1/oar-mir-221.pdf]

miRBase precursor : oar-mir-221
Total read count : 2252
oar-miR-221 read count : 2027
remaining reads : 225

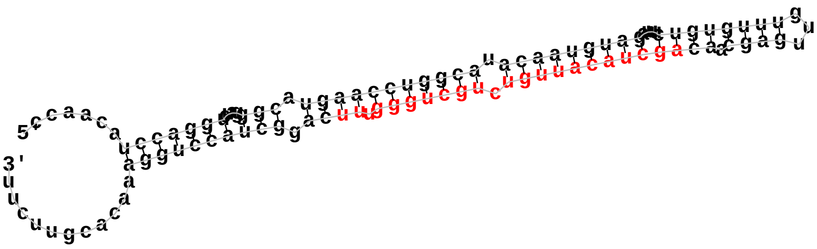

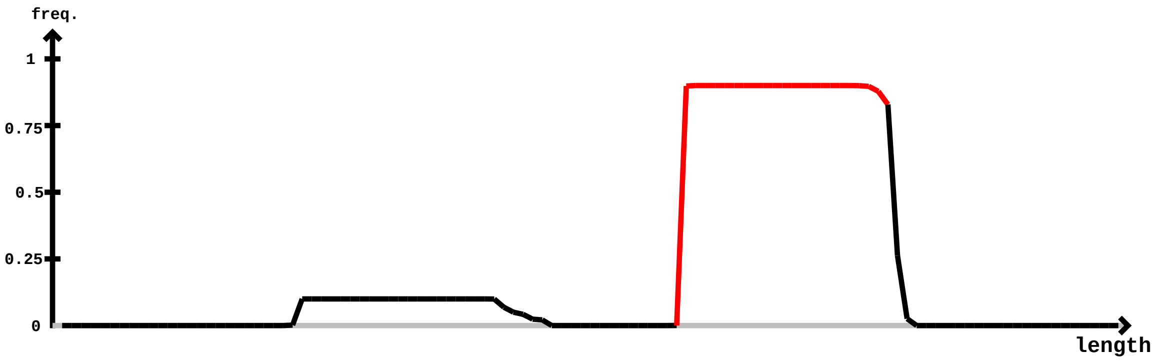

oar-miR-221

5'- ccaacauccaggucuagggcaugaaccuggcauacaauguagauuucuguguuuguugagcaacagcuacauugucugcuggguuucaggcuaccuggaaacacguucuu -3'	exp		
......((((((....(((.(((((((((((.(((((((((....((((((((...)))).))))))))))))).))))))).)))).))))))))))...........	reads	mm	sample
.......................aaccuggcauacaauguagauu...	3	0	seq
.......................aaccuggcauacaauguagauuuc...	1	0	seq
........................accuggcauacaauguagau..	1	0	seq
........................accuggcauacaauguagauu...	66	0	seq
........................accuggcauacaauguagauuu..	42	0	seq
........................accuggcauacaauguagauuuc...	17	0	seq
........................accuggcauacaauguagauuucu..	41	0	seq
........................accuggcauacaauguagauuucug...	6	0	seq
........................accuggcauacaauguagauuucugu..	48	0	seq
....cagcuacauugucugcuggguuu........................	1	0	seq
....agcuacauugucugcugg............................	1	0	seq
....agcuacauugucugcuggg...........................	6	0	seq
....agcuacauugucugcugggu..........................	42	0	seq
....agcuacauugucugcuggguu.........................	110	0	seq
....agcuacauugucugcuggguuu........................	1273	0	seq
....agcuacauugucugcuggguuuc.......................	532	0	seq
....agcuacauugucugcuggguuuca......................	58	0	seq
....gcuacauugucugcuggguu.........................	1	0	seq
....gcuacauugucugcuggguuu........................	3	0	seq
